# Carbon-Based Materials in Combined Adsorption/Ozonation for Indigo Dye Decolorization in Constrain Contact Time

**DOI:** 10.3390/molecules29174144

**Published:** 2024-08-31

**Authors:** Naghmeh Fallah, Ermelinda Bloise, Elisa I. García-López, Giuseppe Mele

**Affiliations:** 1 Department of Engineering for Innovation, University of Salento, Via Monteroni, 73100 Lecce, Italy; naghmeh.fallah@unisalento.it (N.F.); giuseppe.mele@unisalento.it (G.M.); 2 Institute of Atmospheric Sciences and Climate, ISAC-CNR, Str. Prv. Lecce-Monteroni km 1.2, 73100 Lecce, Italy; 3 Department of Biological, Chemical and Pharmaceutical Sciences and Technologies (STEBICEF), University of Palermo, Viale delle Scienze, 90128 Palermo, Italy; elisaisabel.garcialopez@unipa.it

**Keywords:** catalytic ozonation, indigo dye, carbon nitride, multi-walled carbon nanotubes, activated carbon

## Abstract

This study presents a comprehensive evaluation of catalytic ozonation as an effective strategy for indigo dye bleaching, particularly examining the performance of four carbon-based catalysts, activated carbon (AC), multi-walled carbon nanotubes (MWCNT), graphitic carbon nitride (g-C_3_N_4_), and thermally etched nanosheets (C_3_N_4_-TE). The study investigates the efficiency of catalytic ozonation in degrading Potassium indigotrisulfonate (ITS) dye within the constraints of short contact times, aiming to simulate real-world industrial wastewater treatment conditions. The results reveal that all catalysts demonstrated remarkable decolorization efficiency, with over 99% of indigo dye removed within just 120 s of mixing time. Besides, the study delves into the mechanisms underlying catalytic ozonation reactions, elucidating the intricate interactions between the catalysts, ozone, and indigo dye molecules with the processes being influenced by factors such as PZC, pKa, and pH. Furthermore, experiments were conducted to analyze the adsorption characteristics of indigo dye on the surfaces of the materials and its impact on the catalytic ozonation process. MWCNT demonstrated the highest adsorption efficiency, effectively removing 43.4% of the indigo dye color over 60 s. Although the efficiency achieved with C_3_N_4_-TE was 21.4%, which is approximately half of that achieved with MWCNT and less than half of that with AC, it is noteworthy given the significantly lower surface area of C_3_N_4_-TE.

## 1. Introduction

The various processes of the textile industry, such as dyeing, bleaching, printing, and finishing, produce a significant amount of wastewater. This wastewater is not only deeply colored but also laden with chemical oxygen demand (COD), suspended particles, and persistent substances such as heavy metals, artificial dyes, and non-ionic surfactants. The discharge of untreated wastewater into water bodies not only mars their visual appeal but also disrupts aquatic photochemical processes by reducing the transparency needed for light to penetrate. Moreover, these dyes pose a risk of causing cancer and genetic defects, and they can inflict significant harm on human health, potentially leading to disorders of the brain, liver, kidneys, reproductive system, and central nervous system. Therefore, it is crucial for environmental protection to eliminate textile dyes from wastewater [1,2].

Indigo, a widely marketed organic dye, is used for coloring textiles, paper, leather, and plastics, and in specialized areas like food, drugs, cosmetics, and photochemicals. This ancient blue dye remains popular for coloring cotton yarns used in denim and blue jeans production. The demand for indigo and similar vat dyes is growing, with current usage around 33 million kilograms annually. Indigo dye in wastewater is noted for its deep blue hue, which results from dye that does not attach to the fabric during the dyeing process [3,4]. Strategies aimed to remove this color include adsorption, membrane filtration, and coagulation-flocculation, but these can lead to issues such as sludge, membrane blockages, and changes in pollutant phases [5,6]. On the other hand, biological methods using specific bacterial and fungal strains are effective and cost-friendly for color removal, but they are hard to sustain on a large scale in real-world conditions. Recently effective results have been obtained by advanced oxidation technologies (AOTs). These methodologies are often used to mineralize organic pollutants and recalcitrant chemicals usually present in wastewater. AOTs utilize various oxidants such as H_2_O_2_, O_3_, or Fe (II) to produce reactive oxidizing species (ROS) namely ^•^OH, ^•^O_2_^−^, ^1^O_2_, and h^+^ [7]. Some AOTs, such as ozonation, photocatalytic degradation under UV light irradiation in homogeneous or heterogeneous conditions, and the presence of Fenton’s reagent have also been explored as suitable alternatives for decolorizing textile wastewater [8,9,10]. Ozone (O_3_), in particular, is an effective oxidant capable of breaking down the complex structures within dyes [11].

Catalytic ozonation has recently attracted attention as a cutting-edge method for removing organic pollutants from water, even particularly recalcitrant [12,13]. This strategy is gaining research interest because it speeds up the ozonation process and enhances the overall mineralization of the pollutants. Activated carbons (ACs) can enhance the O_3_ degradation to give highly reactive oxidant species (ROS) [14], which play a key role in breaking down and facilitating the mineralization of organic pollutants. Additionally, certain organic compounds that are resistant to degradation through ozonation alone can be effectively oxidized using catalytic ozonation under standard temperature and pressure conditions [15]. A commercially available multi-walled carbon nanotube was effectively employed in catalyzing the ozonation of oxalic acid in an aqueous solution [16]. The mineralization of sulfamethoxazole [17], bezafibrate [18], and erythromycin [19] was improved by using multi-walled carbon nanotubes. Recently advanced oxidation processes utilizing g-C_3_N_4_ catalysts have garnered interest due to their exceptional ability to oxidize and their stable performance. These include Fenton-based reactions, catalytic ozonation, and reactions in the presence of persulfates. In such processes, oxidizing agents such as O_3_ are activated by g-C_3_N_4_ catalysts, leading to the creation of highly reactive species that break down organic pollutants in water [20]. While the addition of carbon materials is known to improve the decomposition of O_3_ in water, the exact mechanism by which they act as initiators, catalysts, or promoters of radical reactions remains unclear.

In many industrial wastewater treatment processes, such as those involving catalytic ozonation, the contact time between the catalyst and wastewater tends to be relatively short. This is primarily due to the continuous flow nature of industrial operations and the necessity to process large volumes of wastewater efficiently. In continuous flow systems, wastewater flows continuously through the treatment process, often at high flow rates to accommodate the volume of wastewater generated by industrial processes. As a result, the residence time of wastewater within the treatment system, including the contact time with the catalyst, is limited [21]. To compensate for the short contact time, industrial wastewater treatment systems often employ strategies to maximize the efficiency of pollutant removal within the available timeframe. This may include optimizing the design of reactors or catalytic systems, enhancing the mixing and dispersion of catalysts within the wastewater stream, and selecting catalyst materials with high activity and selectivity for the target pollutants.

To our knowledge, there is limited information regarding the catalytic ozonation over short contact time for indigo dye removal/bleaching. The primary aim of this study is to assess the catalytic effectiveness of four carbon-based catalysts in the ozonation of potassium indigotrisulfonate (ITS) dye within the constraints of short contact times. Due to the propensity of these materials to adsorb dyes, additional adsorption tests were performed to better understand their role in the ozonation treatment of ITS blue dye.

## 2. Results and Discussion

### 2.1. Material Characterization

**Figure 1:**
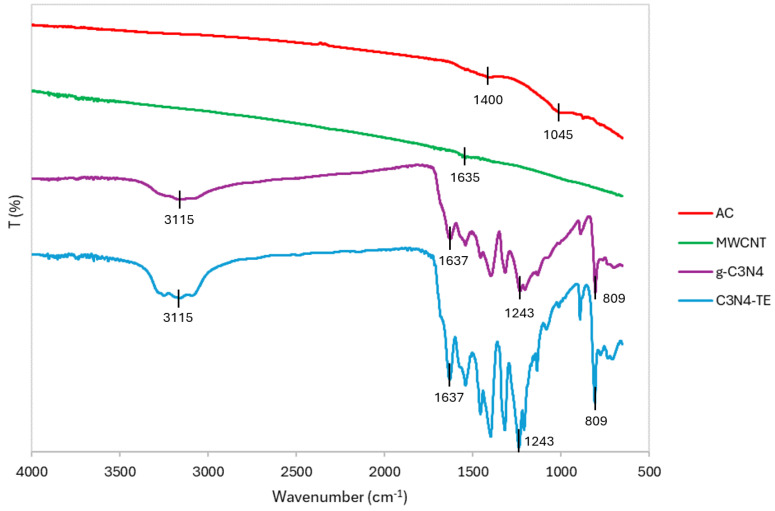
FTIR spectra of AC, MWCNT, g-C_3_N_4_, and C_3_N_4_-TE.

The functional groups of four carbon-based catalysts have been characterized using Fourier Transform Infrared Spectroscopy (FTIR), as illustrated in Figure 1.

In the FTIR spectrum of AC, several notable bands were identified. The band at 1045 cm^−1^ falls within the range associated with –C–O–C stretching vibrations. Additionally, a band observed at 1400 cm^−1^ corresponds to H–O bending vibrations found in phenols and carboxyl groups [22,23]. The FTIR spectrum of MWCNT displays a characteristic peak at 1635 cm^−1^. This peak is attributed to C=C stretching vibrations within the benzenoid rings [24,25]. The presence of this peak suggests that the MWCNT retains the conjugated double-bond structure of the graphene sheets that form the nanotubes. For carbon nitride, both g-C_3_N_4_ and C_3_N_4_-TE exhibit several distinctive peaks in their FTIR spectra. A broad peak around 3115 cm^−1^ is attributed to N–H stretching vibrations, indicating the presence of amine groups. Furthermore, peaks associated with the stretching vibrations of aromatic C–N and C=N bonds in the heterocycles are observed between 1243 and 1637 cm^−1^. These peaks are attributed to the polymeric melon structure of g-C_3_N_4_, which consists of tri-s-triazine units linked by nitrogen atoms. An additional characteristic peak for the s-triazine ring vibrations of g-C_3_N_4_ is observed at 809 cm^−1^. This peak confirms the presence of the triazine ring structure, a fundamental component of g-C_3_N_4_’s framework [26,27,28].

**Figure 2:**
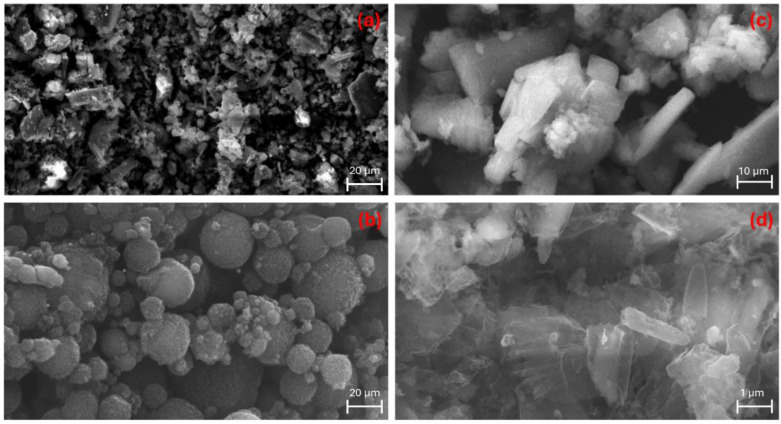
SEM image of AC (**a**); MWCNT (**b**); g-C_3_N_4_ (**c**); and C_3_N_4_-TE (**d**).

The surface morphology of the carbon-based catalysts was analyzed using Scanning Electron Microscopy (SEM). The SEM images of AC, MWCNT, g-C_3_N_4_, and C_3_N_4_-TE at different magnification are shown in Figure 2.

The SEM image of the AC sample (Figure 2a) reveals a heterogeneous surface with areas characterized by rough textures. The AC surface displays a variety of randomly distributed pore sizes, indicating a porous structure essential for adsorption processes. The presence of rough and uneven textures suggests a high surface area [22,23]. The SEM image of MWCNT (Figure 2b) shows the typical morphology of carbon nanotubes. Nanotubes commonly exhibit a tendency to form bundles and become entangled with each other. Nonetheless, the pristine morphology of the CNTs is well-preserved, indicating their structural integrity [24,25]. The SEM image of g-C_3_N_4_ (Figure 2c) reveals the presence of layered structures and stacks, displaying smooth surfaces and irregular shapes. The 2D worm-like layered nanosheet structures are clearly visible, indicating a high degree of crystallinity and a well-defined morphology. The SEM image of C_3_N_4_-TE (Figure 2d) shows a compact structure with large, layered sheet-like surfaces. The modification appears to enhance the structural compactness. The layered morphology can provide extensive surface area and active sites [26,27,28].

**Figure 3:**
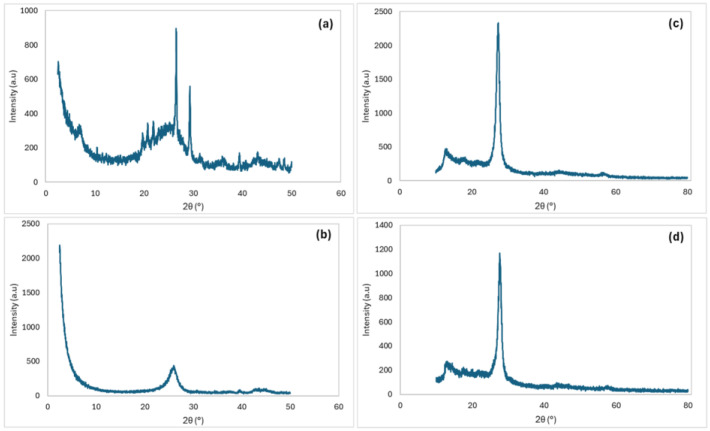
XRD pattern of AC (**a**); MWCNT (**b**); g-C_3_N_4_ (**c**); and C_3_N_4_-TE (**d**).

The X-ray diffraction (XRD) profiles of the carbon-based catalysts provide insights into their crystalline structures (Figure 3).

The XRD profile of the AC sample is shown in Figure 3a. The sample presented a prominent diffraction peak at 2θ = 26.5°, which is indicative of graphitized carbon. However, the peak observed at 2θ = 29° suggests the presence of impurities in the commercial AC samples [22,23]. In the XRD profile of MWCNT (Figure 3b), the peak around 2θ = 26° corresponds to the (002) planes of the graphitic structure within the nanotubes. This peak signifies the graphitic nature of MWCNTs and confirms the presence of well-aligned carbon layers [24,25]. The XRD profiles of g-C_3_N_4_ and C_3_N_4_-TE are depicted in Figure 3c,d. The observed peaks are consistent with the graphitic-C_3_N_4_ layered structure, confirming the successful synthesis of these materials. The prominent peak at 2θ = 27.4° is characteristic of the interlayer stacking of conjugated aromatic C-N heterocycles in g-C_3_N_4_, reflecting its ordered graphitic structure [26,27,28].

### 2.2. Adsorption Studies

Adsorption is a physicochemical process where solid substances attract molecules of gases or solutions to their surfaces. When dissolved molecules in contaminated water come into contact with an adsorbent surface, they adhere both physically and chemically [29].

#### 2.2.1. Effect of Adsorbent Dosage on Decolorization Efficiency

**Figure 4:**
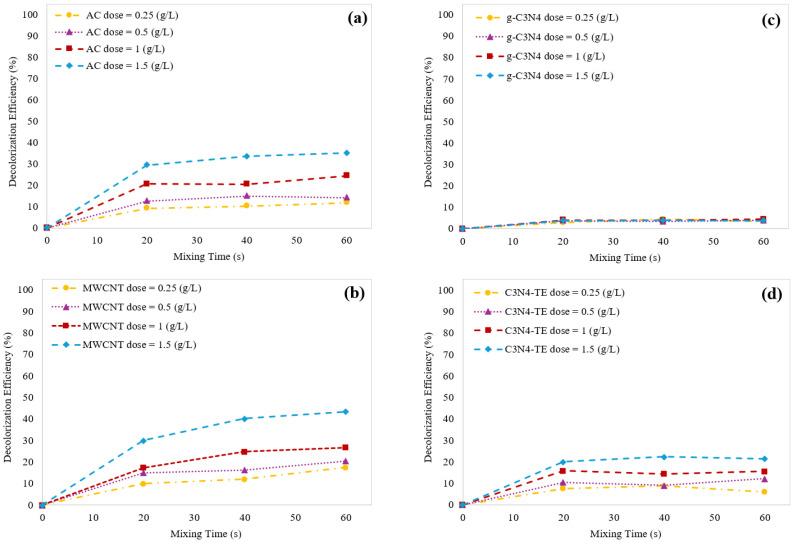
Decolorization efficiency of ITS over time using different doses of AC (**a**), MWCNT (**b**), g-C_3_N_4_ (**c**), and C_3_N_4_-TE (**d**).

The impact of varying adsorbent dosage on the decolorization efficiency of ITS was investigated. The carbon-based materials were used as adsorbents. i.e., AC, MWCNT, g-C_3_N_4_, and C_3_N_4_-TE. All were tested at doses ranging from 0.25 to 1.5 (g/L) shown in Figure 4.

The results revealed that increasing the adsorbent dose led to greater ITS removal and improved decolorization efficiency. While AC and MWCNT showed a gradual increase in efficiency from 0.25 to 1 g/L, the most significant improvement occurred between 1 and 1.5 g/L. Surprisingly, g-C_3_N_4_ exhibited no dose-dependent effect, likely due to its very low surface area.

#### 2.2.2. Adsorption Efficiency Comparison

**Figure 5:**
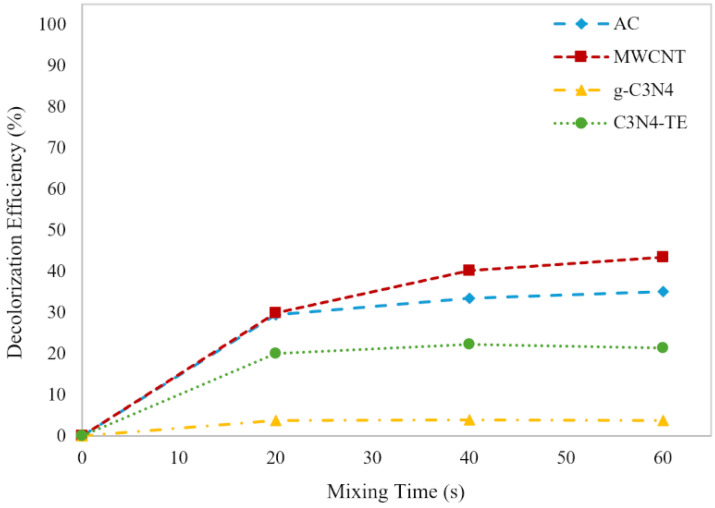
Comparing the decolorization efficiency of ITS over time using AC, MWCNT, g-C_3_N_4_, and C_3_N_4_-TE (1.5 g/L).

A comparison of the ability of the four materials for the efficiency in decolorization of the dye is reported in Figure 5.

Among these, MWCNT exhibited the highest adsorption efficiency, removing 43.4% of the dye absorption at 600 nm. AC performed similarly to MWCNT but was slightly less efficient at longer exposure times.

Interestingly, despite g-C_3_N_4_ and C_3_N_4_-TE being related materials, their adsorption efficiencies differed significantly. This discrepancy can be attributed to their specific surface areas, 6–8 m^2^∙g^−1^ and 170 m^2^∙g^−1^ for g-C_3_N_4_ and C_3_N_4_-TE, respectively.

The outcomes observed with C_3_N_4_-TE were notably intriguing. The decolorization efficiency of ITS using C_3_N_4_-TE was 21.4% during extended mixing time. Although this is approximately half of MWCNT’s efficiency and less than half of AC’s, it’s noteworthy considering that C_3_N_4_-TE possesses a significantly lower specific surface area (170 m^2^∙g^−1^), ca. six times lower than that of AC (1100 m^2^∙g^−1^) and two times lower than that of MWCNT (331 m^2^∙g^−1^), respectively. This insight can be attributed to the surface charge interactions between the solid absorbent surface and the structure of the dye molecule. Experiments were carried out under natural pH conditions, where the C_3_N_4_-TE showed a negative surface charge of conversely, while the dye presented a positive charge of ITS ($PZC=4.63<pH<pK_{a}=11.1$). Under these circumstances, the positively charged dye can be adsorbed on the negatively charged adsorbent surface of the C_3_N_4_-TE leading to an enhanced efficiency of the adsorption and consequently a higher dye removal. This attractive interaction between the dye molecules and the adsorbent surface is crucial for enhancing adsorption efficiency and dye removal for several reasons: (a) The electrostatic attraction between the positively charged dye molecules and the negatively charged adsorbent surface promotes closer contact between them, increasing the likelihood of adsorption. (b) The positively charged dye molecules can bind to the negatively charged sites on the adsorbent surface. This allows for more dye molecules to be accommodated on the surface of the adsorbent material, leading to higher adsorption capacity. (c) Since the adsorbent surface is negatively charged, it can selectively attract positively charged species (like the dye molecules) while repelling negatively charged species. This selectivity enhances the efficiency of dye removal from the solution. As far as the AC material is concerned, the situation is different. The PZC value for AC is approximately 8.5. This means that in a neutral pH environment, the surface of AC becomes positively charged. This positive charge on the surface of the adsorbent material (AC) is the same as the charge of the dye molecules, resulting in a repulsive force between the adsorbent surface and dye molecules which hinders the adsorption process. Even though AC has high surface areas, this repulsion effect can reduce its efficiency in adsorbing dye molecules from the solution. Essentially, the similar charges between the adsorbent surface and the dye molecules can counteract the adsorption process, making the utilization of their high surface area less effective compared to C_3_N_4_-TE.

### 2.3. Catalytic Ozonation Studies

#### 2.3.1. Catalytic Ozonation Efficiency Comparison

**Figure 6:**
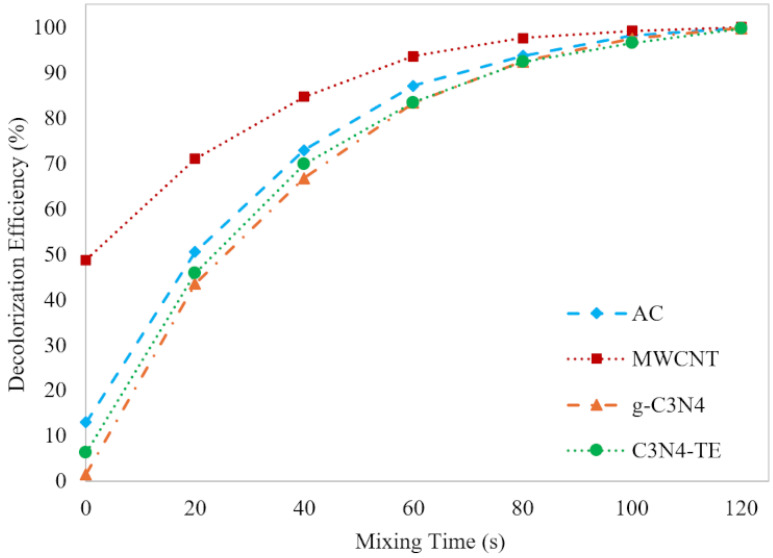
Comparing the catalytic ozonation efficiency for ITS removal over time using 0.25 g/L AC, MWCNT, g-C_3_N_4_, and C_3_N_4_-TE.

The effectiveness of catalytic ozonation in removing the ITS was evaluated in the presence of the four distinct carbon-based materials: AC, MWCNT, g-C_3_N_4_, and C_3_N_4_-TE. Analysis of the decolorization efficiency over various time intervals revealed distinct performance trends for each catalyst. As depicted in Figure 6, the decolorization efficiency surpassed 99% within 120 s of mixing time for all catalysts.

The observed variations in initial efficiency among the catalysts are attributed to the experimental procedure, wherein each catalyst was allowed to establish adsorption/desorption equilibrium with the dye solution before the O_3_ gas injection. This pre-treatment step ensured that the molecule was adsorbed on the surface of the solid catalysts before the ozonation process. Consequently, catalysts such as MWCNT exhibited higher initial efficiencies, benefiting from their rapid adsorption kinetics, while catalysts with slower adsorption kinetics, such as C_3_N_4_-TE and g-C_3_N_4_, demonstrated lower initial efficiencies but exhibited considerable improvements for ITS removal over the catalytic ozonation process.

MWCNT exhibited the highest initial efficiency, reaching complete decolorization within 100 s attributed to its efficient adsorption kinetics. However, beyond the initial phase, C_3_N_4_-TE, g-C_3_N_4_, and AC displayed significant enhancements in decolorization efficiency over time. Particularly, all catalysts showcased sustained catalytic activity, with considerable improvements observed beyond the 60 s.

#### 2.3.2. Catalytic Ozonation Mechanisms

**Figure 7:**
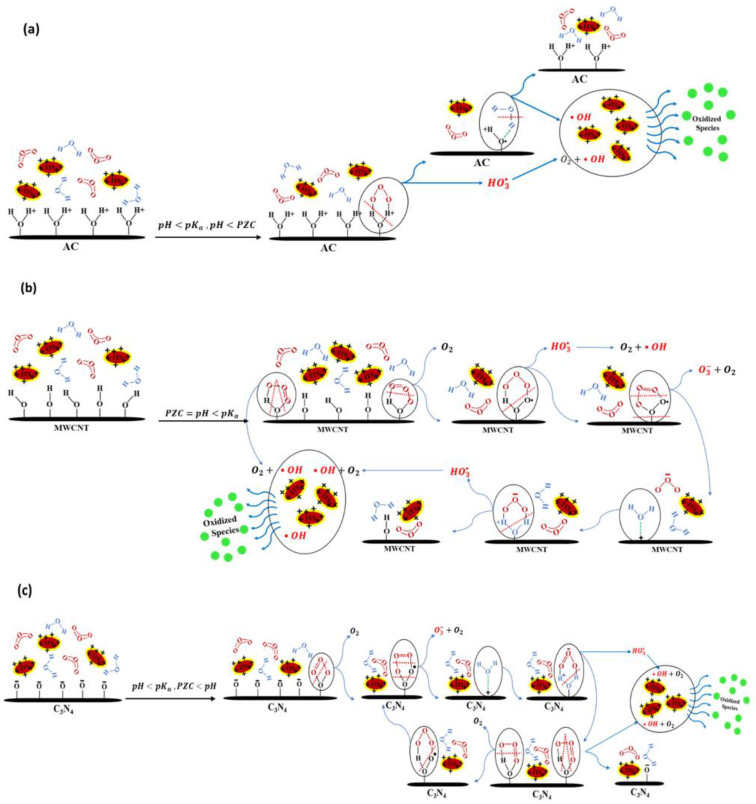
Catalytic ozonation mechanisms proposed for AC (**a**); MWCNT (**b**); g-C_3_N_4_ and C_3_N_4_-TE (**c**).

Generally, the dynamics among the catalyst, pollutant, and O_3_ govern the catalytic ozonation mechanism. Furthermore, the behavior of each of these active components is influenced by various factors, with some exerting more significant effects than others. Based on the existing literature, three primary mechanisms emerge concerning the interaction of catalysts with O_3_ and pollutants: (a) generation of free radicals by O_3_ adsorption onto the catalyst surface and its decomposition. (b) Pollutant adsorption onto the catalyst surface, which is subsequently attacked by O_3_ molecules. (c) Simultaneous adsorption of both O_3_ and pollutants onto the catalyst, leading to their mutual reaction. The proposed mechanisms (Figure 7) can be evaluated by considering the chemical and physical parameters of the catalyst, such as the PZC, the pKa value of the targeted pollutant, and the pH, within catalytic ozonation processes [30].

Experiments were conducted under natural pH conditions (H 7.5–7.6). The pKa value of ITS is 11.1, and the molecule became positively charged under these experimental conditions. Additionally, the different PZC values of each carbon-based material contribute to distinct catalytic ozonation mechanisms for each material.

For AC, with a PZC value of 8.5 higher than the solution’s pH, the surface of the catalyst becomes positively charged. Under these conditions, the adsorption of O_3_ on the AC catalyst’s surface promptly generates HO^•^, crucial for dye oxidation. This O_3_ adsorption leads to the formation of intermediate species $\left({OH}_{3}^{•}\right)$ and additional radicals on the catalyst’s surface. These intermediates transition into reactive HO^•^ radicals and oxygen (O_2_) through an in-situ reaction. Simultaneously, water molecules will adsorb on the catalyst surface due to the existence of radical species on the surface, further generating reactive HO^•^ radicals. However, because ITS is positively charged when pH is less than its pKa, it desorbs from the catalyst. This desorption is driven by repulsive electrostatic forces. As a result, the contaminant is prevented from occupying active surface sites, which limits pore blocking and reduces fouling on the surface (Figure 7a).

MWCNT catalyst with a PZC value of 7 is mostly uncharged in our experimental condition. The uncharged MWCNT catalyst typically has ^•^OH radicals on its surface. Despite being uncharged, these radicals can initiate the decomposition of O_3_. During the O_3_ decomposition reaction chain, after O_3_ adsorbs onto the surface, chemical bonds can stretch and break in various ways. In one scenario, bond breaking directly generates HO^•^ radicals and O_2_. Alternatively, intermediate species such as ${OH}_{3}^{•}$ and $O_{3}^{-}$ may form. The intermediate $({OH}_{3}^{•})\;$then converts into reactive HO^•^ radicals and O_2_ through in-situ reaction. Concurrently, $O_{3}^{-}\;$participates in other chain reactions that ultimately produce HO^•^ radicals (Figure 7b).

In contrast, g-C_3_N_4_ and C_3_N_4_-TE, possessing a PZC value of 4.63, exhibit a negative charge under our experimental conditions. This negatively charged catalyst surface appears to greatly facilitate the adsorption and subsequent breakdown of O_3_. Indeed, O_3_ adsorption on the surface is facilitated, leading to the generation of intermediate species like $O_{3}^{-}$ and ${OH}_{3}^{•}$, which are considered precursors to the highly reactive oxidant species HO^•^. Despite this favorable O_3_ adsorption, pollutant adsorption on the catalyst surface can also occur. The proximity of ITS to the area of HO^•^ radical generation facilitates the rapid oxidation of ITS (Figure 7c).

While some studies have emphasized the role of catalysts’ high specific surface area in their high catalytic effect [31,32], the comparable catalytic efficiency of g-C_3_N_4_ and C_3_N_4_-TE, despite their significantly lower surface areas compared to AC and MWCNT, highlights the significance of the governing catalytic ozonation mechanism in determining efficiency.

#### 2.3.3. Effect of Catalyst Dosage on Decolorization Efficiency

**Figure 8:**
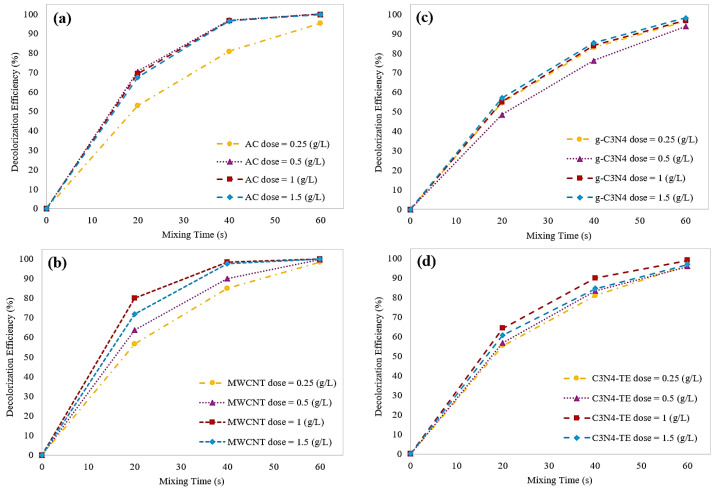
Comparing the catalytic ozonation efficiency for ITS decolorization over time using (**a**) AC, (**b**) MWCNT, (**c**) g-C_3_N_4_, and (**d**) C_3_N_4_-TE.

Our previous catalytic ozonation experiments highlighted that all catalysts showcased considerable improvements observed beyond the 60 s. So, in the pursuit of understanding how catalyst dosage influences the decolorization process of ITS, a series of experiments over 60 s using catalyst dosage ranging from 0.25 to 1.5 g/L have been carried out. Figure 8 depicts the decolorization efficiency of ITS over a 60 s period for each material.

A direct correlation between catalyst dosage and decolorization rate was observed. As the catalyst dosage increased, the number of active surface sites increased and consequently, more O_3_ molecules were effectively transformed into ^•^OH. Specifically, when the catalyst dosage ranged from 0.25 to 1 g/L, the ITS decolorization rate enhanced. However, increasing the catalyst dosage further to 1.5 g/L yielded only marginal enhancements or, in the case of C_3_N_4_-TE, even a slight decrease in the decolorization rate. This intriguing behavior can be attributed to excess ^•^OH generation. At higher catalyst dosages, O_3_ led to the production of an abundance of ^•^OH and paradoxically, this excess of radicals hindered effective consumption during the decolorization process and radicals either underwent recombination reactions or reacted with O_3_ itself.

## 3. Materials and Methods

### 3.1. Materials

In this study, four types of carbon materials were selected. Powder-activated carbon was purchased from Chimica D’Agostino (Bari, Italy) and supplied by AquaSoil s.r.l. (Fasano (BR), Italy). The specific surface area of the activated carbon (AC) used was 1100 m^2^∙g^−1^, with a bulk density of 0.44 g/L. According to existing literature, the point of zero charge (PZC) for this activated carbon is 8.5 [17,33,34].

Multi-walled carbon nanotubes (MWCNTs) were produced by SouthWest NanoTechnologies, Inc. (Norman, OK, USA) and used as received. Previous studies have reported a PZC value of 7 and a surface area of 331 m^2^∙g^−1^ for this multi-walled carbon nanotubes [17,35].

Graphitic carbon nitride g-C_3_N_4_ was used as the precursor for the preparation of thermally etched nanosheets (C_3_N_4_-TE), as previously reported [26,35]. In detail, the bulk carbon nitride (g-C_3_N_4_) sample was synthesized via thermal condensation from melamine. Initially, 10 g of melamine was placed in a ceramic crucible, covered with a lid, and heated in a muffle furnace at a rate of 2 °C per minute until reaching 520 °C. The mixture was then maintained at that temperature for 2 h and gradually cooled down. This resulting g-C_3_N_4_ served as the precursor for the preparation of thermally etched C_3_N_4_ with exfoliated nanosheets (C_3_N_4_-TE). To create C_3_N_4_-TE, 6 grams of g-C_3_N_4_ were ground into a powder, evenly spread at the base of a 14 cm diameter ceramic bowl, and calcined for 2 h in a static air atmosphere at 520 °C using a temperature ramp of 2 °C per minute. The specific surface area of g-C_3_N_4_ is in the range of 6–8 m^2^/g, while C_3_N_4_-TE has a specific surface area of 170 m^2^∙g^−1^. Additionally, their respective PZC values are 4.63 [36,37].

The ITS dye was purchased from Sigma-Aldrich and utilized without any additional purification. The ITS solution was prepared by dissolving it in tap water.

O_3_ gas was produced by oxygen from air by an ozonator (ECS MADE IN EU, Ref: ZY-H1159), with a flux of 13–15 g∙h^−1^, using the O_3_ generation method of Corona discharge in a closed chamber.

### 3.2. Characterization Techniques of the Catalysts

Different techniques were used to characterize the carbon-based catalysts. The identification of the functional groups was carried out by a Jasco FTIR-660 PLUS spectrometer (Jasco, Easton, MD, USA) fitted with a micro-ATR crystal sampler to confirm their chemical structure.

Morphological analyses were performed by means of a Scanning Electron Microscope (SEM) by ZEISS (Oberkochen, Germany), EVO 40.

X-ray power diffraction (XRD) was performed on the carbon-based materials, with a Rigaku Ultima+ model diffractometer (Rigaku, Tokyo, Japan). The patterns were obtained by Cu Kα radiation (λ = 0.15406 nm), with a focus size of 0.4 × 12 mm, a rated tube voltage 40 kV, and a goniometer radius of 285 mm.

### 3.3. Experimental Procedures

#### 3.3.1. Dye Solution Preparation

The ITS solution utilized in this study was prepared by dissolving the solid chemical in tap water to achieve a concentration of 80 mg/L. This concentration was chosen to mimic the high levels of ITS reported in wastewater, reaching up to 2000 mg/L [38]. By testing this initial concentration of the dye, the study aimed to evaluate the contribution of catalysts in its degradation, particularly considering the rapid discoloration of the dye induced by O_3_ gas due to its higher reactivity compared to the aqueous phase.

The dye concentration was measured by using a UV-visible spectrophotometer (V-660 Jasco) by the absorbance of the dye at 600 nm, corresponding to the maximum absorption of ITS.

#### 3.3.2. Short-Term Adsorption Experiment

Short-term adsorption experiments were performed in a beaker using 40 mL of the prepared ITS solution. Carbon-based materials ranging from 0.25 g/L to 1.5 g/L were added to the solution. Post-treatment samples were collected at 20 s intervals over 60 s of stirring time and analyzed for ITS absorbance measurements. Stirring was facilitated using a magnetic stir bar at atmospheric pressure and room temperature, followed by filtration through a 0.2 µm polypropylene membrane. The filtered solution was subsequently analyzed with a UV-visible spectrophotometer at 600 nm, the wavelength corresponding to the maximum absorption of ITS.

#### 3.3.3. Catalytic Ozonation Process

The catalytic ozonation process was carried out in a beaker with 40 mL of the ITS solution. Initially, 0.25 g/L of catalyst was added to the solution and stirred for 30 min to achieve the adsorption/desorption equilibrium before the O_3_ gas injection. Prior to O_3_ gas treatment, the gas flow was stabilized for 2 min. Subsequently, O_3_ gas was injected into the samples, and at specific time intervals (20, 40, 60, 80, 100, and 120 s), samples were collected and sodium thiosulfate (0.3 M) was applied to quench the residual O_3_.

In order to study the effect of catalysts doses, carbon-based catalysts ranging from 0.25 g/L to 1.5 g/L were added to the solution. Post-treatment samples were collected at 20 s intervals over 60 s of stirring time and analyzed for ITS absorbance measurements. In this case, the initial adsorption effects are accounted for before evaluating the catalytic activity by normalizing the data with respect to the absorbance value after the adsorption process.

#### 3.3.4. pKa Determination of ITS

The pKa of ITS was determined using a UV-spectrophotometric method. A range of solutions with pH values from 2 to 12 were prepared using appropriate buffers, and the pH of each solution was verified using a calibrated pH meter. UV-visible absorption spectra of ITS solutions at each pH were recorded to the maximum absorption of ITS (600 nm). The absorbance data obtained at each pH were used to construct a titration curve, with absorbance plotted against pH. The pKa value of 11.1 was determined from the inflection point of the titration curve, representing the pH at which there is an equal concentration of the protonated and deprotonated forms of the dye [39,40].

## 4. Conclusions

This study has provided an assessment of catalytic ozonation as a viable treatment method for the removal of indigo blue dye, focusing specifically on the performance of four carbon-based catalysts, i.e., AC, MWCNT, g-C_3_N_4_, and C_3_N_4_-TE. The efficiency of catalytic ozonation within short contact times, representative of real-world industrial wastewater treatment conditions, was thoroughly investigated.

The results obtained from this study underscore the remarkable decolorization efficiency achieved by all chosen catalysts, with over 99% of dye bleached within just 120 s of mixing time. This highlights the potential of catalytic ozonation as a rapid and effective method for dye removal.

Furthermore, this study provides a comprehensive exploration of the mechanisms driving catalytic ozonation reactions. It offers detailed insights into the complex interactions that occur between the catalysts, ozone, and indigo dye molecules. These interactions are shaped by key factors, including PZC of the catalysts, pKa of the dye, and the pH of the reaction environment. By examining how these variables influence the behavior of the system, the study sheds light on the fundamental processes that govern the effectiveness of catalytic ozonation in pollutant degradation.

Additionally, experiments examining the adsorption behaviors of indigo dye onto the surfaces of the catalyst materials provided valuable insights into their impact on the catalytic ozonation process. Notably, MWCNT exhibited the highest adsorption efficiency, removing 43.4% of the dye. While the efficiency achieved with C_3_N_4_-TE was comparatively lower at 21.4%, it is noteworthy considering the significantly lower surface area of C_3_N_4_-TE compared to MWCNT and AC. Furthermore, the proposed mechanisms of catalytic ozonation processes shed light on the intricate interactions between the catalysts, O_3_, and indigo dye molecules. Overall, this study contributes to the understanding of catalytic ozonation as a promising approach for indigo dye removal in industrial wastewater treatment. The findings underscore the importance of catalyst selection and experimental pH, providing valuable insights for the optimization of catalytic ozonation processes in real-world applications. Further research in this area holds promise for advancing sustainable solutions for wastewater treatment and environmental protection.

## Data Availability

The original contributions presented in the study are included in the article, further inquiries can be directed to the corresponding author.
